# Effects of different transcranial direct current stimulation protocols on visuo-spatial contextual learning formation: evidence of homeostatic regulatory mechanisms

**DOI:** 10.1038/s41598-020-61626-7

**Published:** 2020-03-12

**Authors:** Paolo A. Grasso, Elena Tonolli, Carlo Miniussi

**Affiliations:** Centre for Mind/Brain Sciences - CIMeC, University of Trento, Rovereto, TN Italy

**Keywords:** Spatial memory, Visual system

## Abstract

In the present study we tested the effects of different transcranial direct current stimulation (tDCS) protocols in the formation of visuo-spatial contextual learning (VSCL). The study comprised three experiments designed to evaluate tDCS-induced changes in VSCL measures collected during the execution of a visual search task widely used to examine statistical learning in the visuo-spatial domain. In Experiment 1, we probed for the effects of left-posterior parietal cortex (PPC) anodal-tDCS (AtDCS) at different timings (i.e. offline and online) and intensities (i.e. 3 mA and 1.5 mA). The protocol producing the more robust effect in Experiment 1 was used in Experiment 2 over the right-PPC, while in Experiment 3, cathodal-tDCS (CtDCS) was applied over the left-PPC only at a high intensity (i.e. 3 mA) but varying timing of application (offline and online). Results revealed that high intensity offline AtDCS reduced VSCL regardless of the stimulation side (Experiment 1 and 2), while no significant behavioral changes were produced by both online AtDCS protocols (Experiment 1) and offline/online CtDCS (Experiment 3). The reduced VSCL could result from homeostatic regulatory mechanisms hindering normal task-related neuroplastic phenomena.

## Introduction

Transcranial electrical stimulation (tES) is a non-invasive brain stimulation technique extensively used in cognitive neuroscience to produce transient modifications of brain activity. Among the various existing tES protocols, transcranial direct current stimulation (tDCS) is doubtless the most known and a wealth of works have demonstrated its capability to modulate neural responses and to shape behaviour^1–4^. It consists in the application of weak direct electrical currents (mostly, 1 or 2 mA) for a few minutes (usually not more than 30 minutes) via a pair of electrodes positioned on the scalp. The electrical field generated by the current is capable to modulate membrane permeability and post-synaptic neuronal activity in both excitatory and inhibitory fashion. Because of single neuron registrations, we know that anodal-tDCS (AtDCS) induces an increase (depolarization) and cathodal-tDCS (CtDCS) causes a decrease (hyperpolarization) in permeability of the of neuronal membrane potential of stimulated neurons^5,6^. In the long run (i.e., after effects), the two processes are thought to promote neuroplasticity through the induction of long-term potentiation (LTP) and long-term depression (LTD) -like effects^1,7–9^.

At the behavioural level, using the stimulation-dependent model, it is generally expected that AtDCS applied during the execution of a task leads to enhanced behavioural performances on the task while the opposite is expected for CtDCS. These assumptions are primarily related to the abovementioned effects of tDCS on membrane permeability. Nevertheless, this model directly and simplistically associates the effects at the neuronal level to the effects at the behavioural level. Moreover, this description clearly represents an approximation mainly assuming the brain as a passive system. The coarseness of this picture has been confirmed by several studies revealing the non-linearity of tDCS-induced effects, an evidence suggesting that much remains to be understood about tDCS-brain interactions^3^. It is plausible that, at least part, of the reported non-linear effects could arise from a limited comprehension of the interactions occurring between tDCS-induced modifications and the state of the system or its governing mechanisms^10–12^. Accordingly, it has been demonstrated that the effects of tDCS can be greatly influenced by the ongoing brain activity given that the level of engagement during the execution of an experimental task can even reverse the expected effects of the stimulation^13–16^. In addition, we also know that the brain is anything but a passive system and is endowed with adaptive mechanisms enabling it to dynamically react to external inputs or changes in the environmental demands. Homeostatic regulatory mechanisms are an evidence of this. They consist in the ability of the brain to dynamically stabilize its own activity around a physiologically reasonable range in order to prevent itself from hyper-/hypo-excitability^17^. In this view, when within the same cortical area/network a prolonged tES-induced brain activity (increase/decrease) is associated with a task-induced (increase) brain activity (e.g., during learning protocols), we could expect the two processes to dynamically interact. Previous works confirmed this idea and provided wide behavioural and neurophysiological support for the presence of homeostatic regulatory principles in the human brain. Reported evidence mainly pertain the effects produced by tDCS applied over the primary motor cortex in close temporal sequence with a task involving motor learning^14,18–20^. For example, Kuo and colleagues demonstrated that prior application of AtDCS (1 mA) reduced implicit motor learning in a serial reaction time task but left performance unchanged in a simple reaction time task^18^. Amadi and colleagues reported that AtDCS (1 mA) applied before a motor task decreased learning rates and increased GABA_A_ activity as revealed by short interval intracortical inhibition protocol^19^. Stagg and colleagues found that AtDCS (1 mA) applied during a motor learning task enhanced the learning process while the same stimulation protocol applied before the task, reduced learning^20^. Similarly, Bortoletto and colleagues observed that AtDCS (1.5 mA) facilitated motor learning but only when applied in association with a slow motor practice, not producing learning on its own. With the same protocol, learning was inhibited when AtDCS was applied in association with a fast motor practice that usually generate learning on its own by increasing cortical excitability^14^. Interestingly, even reversing the order of tES application and motor practice produced results consistent with mechanisms of homeostatic regulation as revealed by Cantanero and colleagues who reported an occluded motor learning when AtDCS (1 mA) was applied after a sequential visual isometric pinch task^21^. What is less documented is whether similar regulatory mechanisms are retrievable also outside the motor cortex and/or primary sensory areas. Further, it is not clear whether the abovementioned interactions between tES- and task-related brain activity occur also when the learning paradigm involves a more distributed network of cortical activity. For instance, during visuo-spatial statistical learning where a complex interplay between primary and higher order areas is necessary for the learning to occur^22–24^. Finally, the effects produced by stimulation intensities exceeding the standard 1–2 mA has been rarely tested in the literature casting out the possibility to fully understand whether increased/decreased learning could be obtained with increased stimulation intensities.

In the present study, we developed three separate experiments in order to test the effects produced by different tDCS protocols (varying timing, intensity and polarity of application) on visuo-spatial contextual learning (VSCL). In Experiment 1, left-posterior parietal cortex (PPC) AtDCS (i.e., target electrode on left PPC and return electrode on right arm) applied at different timings (offline/online) and intensities (3 mA and 1.5 mA) was associated with the execution of a VSCL task widely used in previous studies examining statistical learning in the visuo-spatial domain^25^. Based on previously described works on the motor domain, we expected AtDCS applied before the task (offline) to significantly interfere with VSCL on a stimulation intensity gradient providing evidence of homeostatic regulatory principles in the PPC. Furthermore, we expected AtDCS applied during the task (online) to potentially facilitate learning, based on intensity and individual ability to perform the task. Experiment 2 was post-hoc designed to control whether results obtained from Experiment 1 could be explained in the framework of a balance between left and right PPC activity. For this reason, the stimulation protocol that produced the more robust effect was used with a reversed electrodes montage (i.e., target electrode on right-PPC and return electrode on left arm). Finally, in Experiment 3, offline and online 3 mA CtDCS protocols (i.e., target electrode on left PPC and return electrode on right arm) were used to test the conceivable effects of neuronal excitability reduction on task performance. In this case, we expected offline CtDCS to produce facilitatory effects on VSCL, while online CtDCS to significantly interfere with VSCL.

## Methods

### Preregistration

The present study was preregistered prior to data collection at the Centre for Open Science through the Open Science Framework and is visible at the present address: https://osf.io/rwx29.

The original preregistered project comprised additional data and statistical analysis that are not included in the main body of the manuscript. These elements are however retrievable in the Supplementary Information (Appendix A–C). Moreover, both Experiment 2 and Experiment 3 were not included in the preregistration since they have been post-hoc designed to explore additional aspects related to results obtained in Experiment 1. To ease the identification of those analyses originally reported in the preregistration form (*confirmatory analyses*) from those not reported (*exploratory analyses*), an indication adhering to the following code has been added alongside each result both in the Manuscript and in the Supplementary Information. ^(CF)^ = Confirmatory Analyses; ^(EX)^ = Exploratory Analyses.

### Participants

One hundred and fourteen healthy participants were screened for the presence of any contraindications to the use of tES^26^ and only those without contraindications took part to the study. This procedure led to the selection of one hundred and twelve participants (mean age: 22.6 years, sd: 3.2 years, 56 males) all naïve to the purpose of the study, right handed (as assessed by the Edinburgh Handedness Inventory^27^) and with normal or corrected to normal visual acuity. Participants were pseudo-randomly assigned to one of the different electrical stimulation protocols chosen for the present study (see *Transcranial Direct Current Stimulation* section below). This resulted in the selection of 14 participants (7 males) per stimulation group.

Before taking part to the experiment, all participants were informed about the procedures of the study and provided written informed consent in accordance with the Declaration of Helsinki. The study was approved by the University of Trento Human Research Ethics Committee and was carried out in accordance with approved guidelines.

### Experimental Task

The visual task used in the present experiment was an adapted version of the spatial contextual cueing paradigm elaborated by Chun and Jiang^25^. The task was to search for a rotated “T” (90° clockwise or 90° counterclockwise) target stimulus amongst eleven heterogeneously rotated “L” distractors and to report target stimulus orientation by pressing “-“ (counterclockwise) or “z” (clockwise) keys on the keyboard.

**Figure 1:**
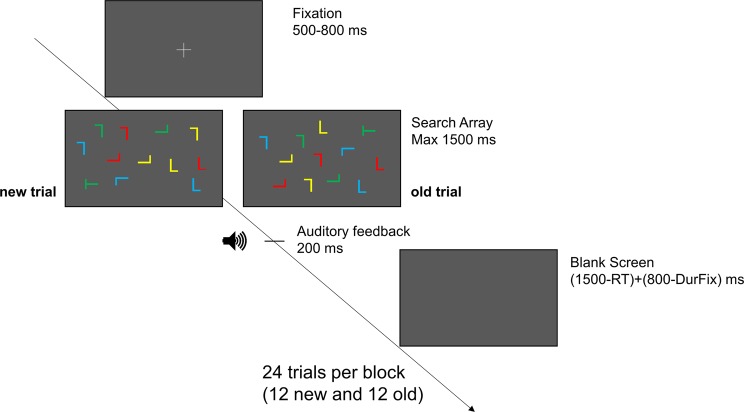
Trial structure. Each trial began with a fixation cross (jittered time between 500 and 800 ms) followed by the presentation of a search array (max 1500 ms). Participants were instructed to search for a target stimulus (rotated T) and to report its rotation by pressing “-” (target rotated counterclockwise) or “z” (target rotated clockwise) keys on the keyboard. Each response was followed by an auditory feedback (200 ms) that varied based on participants’ response (i.e., hit: 800 Hz; incorrect: 200 Hz; miss: 500 Hz). The search array disappeared whenever a response was provided and was followed by a variable duration blank black screen ensuring a constant trial duration regardless of individual response speed. Search arrays could be either “new” or “old”. In *new* arrays, stimuli were randomly displaced on the monitor, while *old* arrays consisted in the presentation of pre-determined arrangements of stimuli. Each *new* search array was presented once in the entire experiment, while each *old* search array was repeated once in each block. Each block consisted in the presentation of 24 trials (12 *new* and 12 *old*) and the whole experiment comprised of 13 blocks.

Each trial started with a fixation cross at the center of the screen (jittered time between 500 and 800 ms) followed by the presentation of the search array. Participants had to report the orientation of the target stimulus within 1500 ms. Each response was followed by an auditory feedback (200 ms) that varied based on response provided (i.e., hit: 800 Hz; incorrect: 200 Hz; miss: 500 Hz). The search array disappeared whenever participants provided a response and was followed by a blank black screen (variable duration) which ensured a constant trial duration regardless of individual RTs. If no response was provided within 1500 ms, a new trial began (see Fig. 1 for task design and timeline).

Each block consisted in the presentation of 24 trials. The whole experiment comprised of 13 experimental blocks (~15 minutes) plus 3 training blocks (~4 minutes) performed before the experiment which served to get participants familiarized with the task.

### Apparatus and stimuli

Participants seated in a dimly lit and sound attenuated room with their head placed on a chinrest at 57 cm from a LED monitor (24 inches, refresh rate: 60 Hz, 1920 × 1200-pixel resolution).

In search displays, stimuli could appear within an invisible array of 8 × 6 grids (27 × 20.5 degrees of visual angle), were heterogeneously colored (red, blue, green and yellow) and were presented amongst a black background. Search arrays could be either “new” or “old” (12 *new* arrays and 12 *old* arrays per block). In *new* arrays, the position, the colour and the rotation of the items were pseudo-randomly assigned. Target stimulus could appear at the centre of one of twelve predetermined grids, could have a random rotation (90° clockwise or counterclockwise) and a random color (red, green, blue or yellow). Each distractor could appear in one of the remaining grids (jittered position within grid’s possible coordinates), could have a random rotation (0°, 0°-horizontally flipped, 90°, 90°-horizontally flipped, 180°, 180°-horizontally flipped, 270°, 270°-horizontally flipped) and a pseudo-random colour. In *old* arrays, the position, the colour and the orientation of items were a priori defined within a set of twelve predetermined arrangements that were repeated across blocks. Only rotation of the target stimulus (90° clockwise or 90° counterclockwise) was randomly varied. In both *new* and *old* trials, an equal number of red, blue, green and yellow items was presented for each search array and an equal number of targets was presented for each colour at the end of each block. Furthermore, in order to control for any effect of proximity to the fixation cross, the eccentricities of target locations were balanced across *new* and *old* arrays.

Training blocks performed before the experiment consisted in the sole presentation of *new* trials as the purpose was to get participants familiarize with the task.

### Transcranial direct current stimulation

Electrical stimulation (3 or 1.5 mA) was delivered by a battery-driven DC stimulator (EMS, BrainStim) via two rubber electrodes inserted in saline-soaked sponges. The target electrode size was 5 × 5 cm while the return electrode size was 6 × 7 cm. To reduce skin impedance, electro-conductive gel (Easycap, SuperVisc Gel) was applied under the sponges and impedance levels were kept below ~5 kΩ. Electrodes were kept in place with the use of elastic bands, which ensured a homogeneous pressure on the electrode surface and minimized the probability of drops in current density^28,29^. In real tDCS protocols, current ramped up and down over the first and last 10 s of stimulation and was applied for 15 minutes. In the control sham tDCS protocol, the stimulation was applied only in the first and last 20 s (i.e., 10 s ramped up and 10 s ramped down) in order to blind participants to the stimulation manipulation. Half of the participants included in the sham tDCS protocol received a current ramping to 3 mA while the other half received a current ramping to 1.5 mA.

In Experiment 1, five different stimulation protocols were administered (P3 as in the 10/20 EEG system - 3 mA offline AtDCS, P3 - 3 mA online AtDCS, P3 - 1.5 mA offline AtDCS, P3 - 1.5 mA online AtDCS and Sham), while one stimulation protocol was included in Experiment 2 (P4 as in the 10/20 EEG system - 3 mA offline AtDCS) and two in Experiment 3 (P3 - 3 mA offline CtDCS, P3 - 3 mA online CtDCS). Current densities were 0.12 mA/cm^2^ and 0.06 mA/cm^2^ for the 3 mA and 1.5 mA protocols respectively.

**Figure 2:**
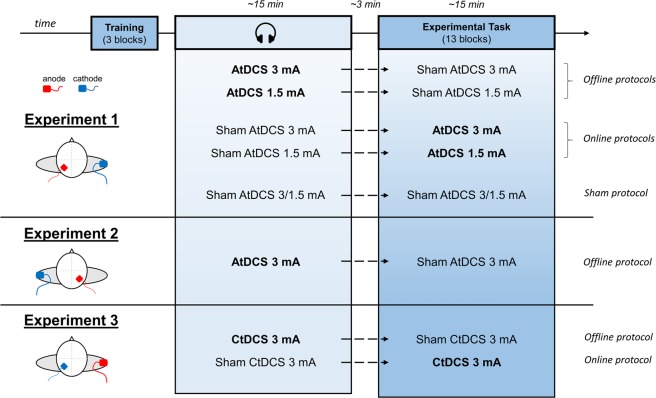
Experiment structure and tDCS application. Each participant was assigned to one of the eight stimulation protocols (five for Experiment 1, one for Experiment 2, and two for Experiment 3). Offline protocols consisted in the application of tDCS before task execution while participants listened to an audiobook whereas online protocols consisted in tDCS applied during task execution. Furthermore, to blind with respect to the timing of the stimulation, participants assigned to the offline protocols also received a sham stimulation during task execution whereas participants assigned to the online protocols received a sham stimulation before task execution while listening to the audiobook. Three training blocks, in which only *new* search arrays were presented, were initially performed with the scope to get participants familiarized with the task. On the left: flight view of tDCS montages used for each experiment. In Experiment 1 anode (red) was positioned on P3 (10/20 EEG system) while cathode (blue) was positioned on the upper part of the right arm. In Experiment 2 a flipped electrodes montage was used with anode placed on P4 and cathode on the upper part of the left arm. In Experiment 3 anode was placed on the upper part of the right arm and cathode on P3.

In the offline protocols, real stimulation was applied before the execution of the task while participants listened to an audiobook, whereas in the online protocols real stimulation was applied during the execution of the task. Furthermore, in order to blind participants with respect to the timing of the real stimulation (i.e., offline/online), a sham stimulation was also administered before or after the real stimulation (i.e., participants assigned to the offline protocols received a sham stimulation during task execution while participants assigned to the online protocols received a sham stimulation during the audiobook; Fig. 2). Participants assigned to the sham stimulation protocol received two identical sham stimulations.

In Experiment 1, the target electrode was placed over P3 corresponding to the inferior part of the left PPC^30^, while the return electrode was positioned in the upper part of the right arm. We chose to stimulate PPC as it is considered one of the key regions involved in the formation of visuo-spatial contextual memories^23,24,31–33^. A reversed electrodes montage was used in Experiment 2 in which the target electrode was placed over P4 and the return electrode was positioned in the upper part of the left arm. In Experiment 3, electrodes position was the same as Experiment 1 but with an inverted polarity.

At the end of all experiments, participants completed a questionnaire about the sensations experienced during the tES in which they were asked to give a score from 0 (none) to 4 (strong) to rate their perception of tES-induced Irritation, Pain, Burning, Heat, Itch, Iron Taste and Fatigue^26,29^. The questionnaire comprised of two identical sections: the first referring to the offline stimulation (real or sham) and the second referring to the online stimulation (real or sham).

### Analysis

VSCL scores (i.e., RTs on *new* trials minus RTs on *old* trials) were used as the main outcome measure. Only hit trials and trials with VSCL scores within 3sd from the individual mean were considered in the analysis. For each experiment, data were analysed using a mixed design analysis of covariance (ANCOVA) which compared the effects of different stimulation protocols on VSCL scores after controlling for general task performance (covariate factor; i.e., mean accuracy scores on *new* trials). The rationale of using ANCOVA arose from the significant correlation found between VSCL scores and mean accuracy scores on *new* trials which suggested a possible influence of general task performance on the main outcome measure (see *Multivariate Outliers’ Detection* section below). In Experiment 2 and Experiment 3, results obtained from the real stimulation protocols were compared with those obtained from the Sham protocol from Experiment 1. Further analyses comprised mixed design analyses of variance (ANOVA) of the RTs and the accuracy scores on *new* trials which were performed for control purposes. Fisher’s Least Significant Difference (LSD) method was used to test the limited number of contrasts of interest (i.e., compare performances of groups receiving real stimulation with the performance of the group receiving sham stimulation). For all the other comparisons, Bonferroni correction was applied. Data from the sensations induced by tDCS were analysed using appropriate non-parametric statistics. A p-value <0.05 was considered significant for all statistical analyses.

## Results

### Multivariate outliers’ detection

^(EX)^ A correlation analysis revealed a significant correlation between general task performance (as assessed by mean accuracy scores on *new* trials) and VSCL scores (*r*(111) = 0.271, *p* = 0.004) suggesting that the main outcome measure (i.e., VSCL scores) could be influenced by participants’ individual ability to perform visual search.

For this reason, a multivariate outliers’ detection procedure combining individual general task performance (independent variable) and VSCL scores (dependent variable) was used to exclude those participants who had performance greatly departing from the average distribution of the group^34^. Specifically, participants who had values of Cook’s distance greater than 1 were excluded. This resulted in the exclusion of one participant initially assigned to the P3 - 3 mA offline AtDCS stimulation group.

### Experiment 1 - left-PPC AtDCS varying timings and intensities

**Figure 3:**
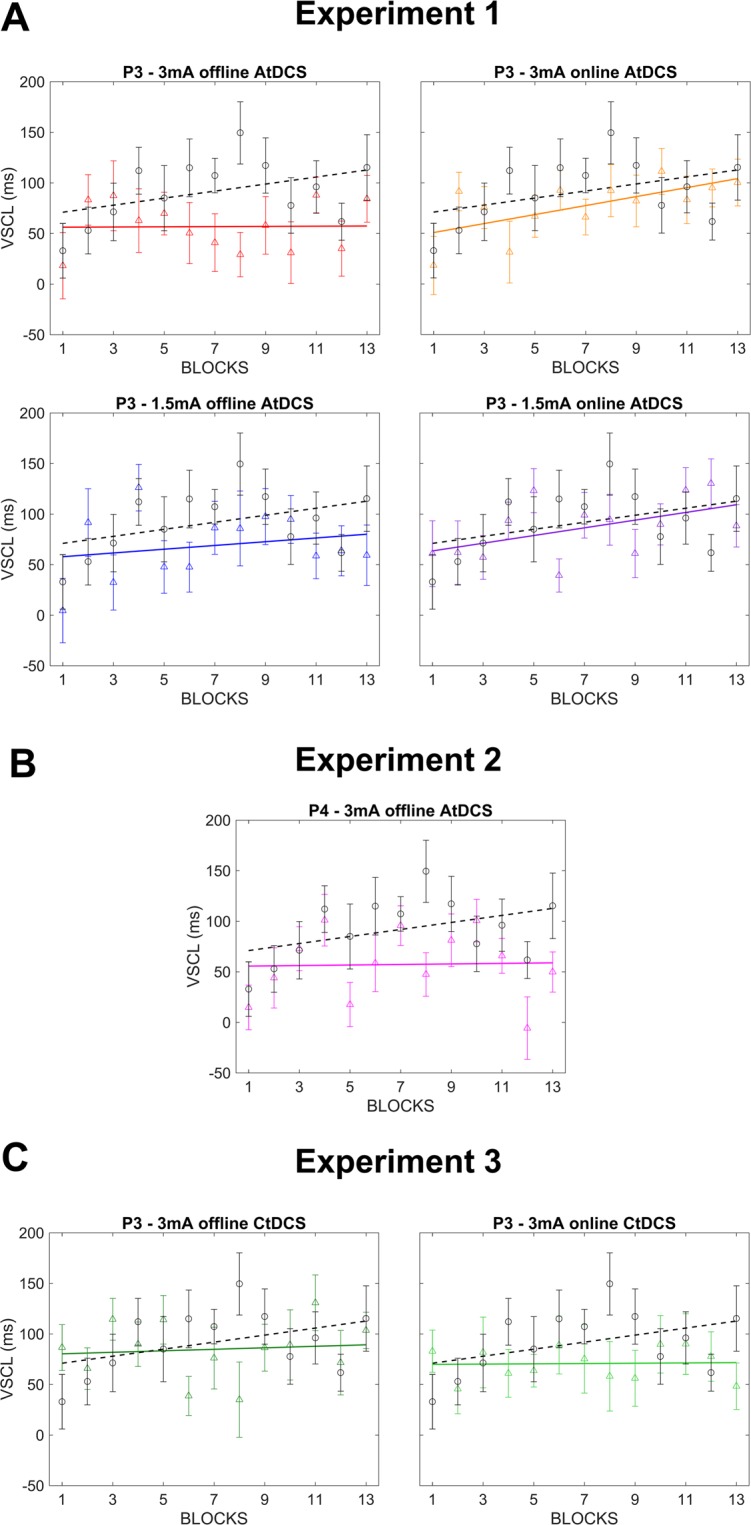
Linear fits of VSCL scores (i.e., RTs on *new* trials minus RTs on *old* trials) across blocks for each tDCS protocol administered. Each graph depicts results obtained by one stimulation protocol (coloured solid line) against the sham control protocol (black dotted line). **A**. Results from Experiment 1 tDCS protocols. **B**. Results from Experiment 2 tDCS protocol. **C**. Results from Experiment 3 tDCS protocols.

**Figure 4:**
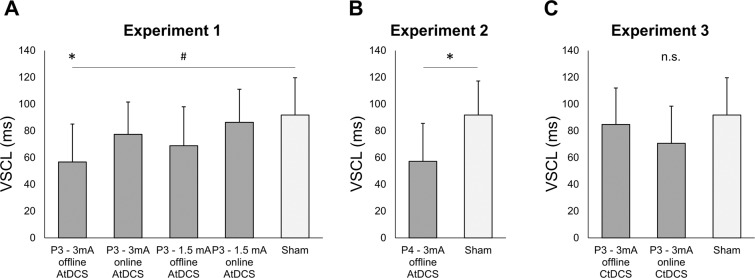
Average VSCL scores (i.e., RTs on *new* trials minus RTs on *old* trials) for tDCS protocols administered in Experiment 1 (**A**), Experiment 2 (**B**) and Experiment 3 (**C**). Symbols depicts significant comparisons (* = p < 0.05; # = p < 0.08).

^(EX)^In order to control whether differences in VSCL scores could be attributable to between groups differences in general task performance, two 5 × 13 mixed ANOVAs with the between factor Stimulation (5 levels) and the within factor Blocks (13 levels) were performed on accuracy scores and RTs to *new* trials. Both analyses revealed a main effect of Blocks (accuracy: *F*(12, 768) = 4.050; *p* < 0.001; *ƞ*_*p*_^2^ = 0.06; observed power = 0.99; RTs: *F*(12, 768) = 4.22; *p* < 0.001; *ƞ*_*p*_^2^ = 0.06; observed power = 0.99) explained by enhanced accuracy scores and a significant reduction of RTs as the task progresses. No main effect of Stimulation (accuracy: *F*(4, 64) = 0.576; *p* = 0.681; *ƞ*_*p*_^2^ = 0.03; RTs: *F*(4, 64) = 0.70; *p* = 0.595; *ƞ*_*p*_^2^ = 0.04) and Blocks*Stimulation interaction (accuracy: *F*(48, 768) = 0.965; *p* = 0.542; *ƞ*_*p*_^2^ = 0.06; RTs: *F*(48, 768) = 1.13; *p* = 0.257; *ƞ*_*p*_^2^ = 0.06) was revealed by the analysis suggesting that general task performances were correctly equated between groups (see also Appendix A in the Supplementary Information for further control analyses on this aspect).

^(EX)^Given the strong dependency of tDCS effects on the neural activity level induced by the task^13–16^, an exploratory additional analysis was conducted after dividing participants into “low performers” and “high performers” based on group accuracy scores’ median values on *new* trials (Table 1). A 5 × 2 × 13 mixed ANOVA with the between factors Stimulation (5 levels) and Performance (2 levels: low performers and high performers) and the within factor Blocks (13 levels) was performed on VSCL scores. In accordance with previous analysis (see above ANCOVA), results revealed a main effect of Stimulation (*F*(4, 59) = 2.854; *p* = 0.031; *ƞ*_*p*_^2^ = 0.16; observed power = 0.74) that was explained by a reduction of VSCL scores in the two offline AtDCS groups with respect to the Sham group (3 mA: *p* = 0.006; 1.5 mA: *p* = 0.063). As expected, a main effect of Performance was evident (*F*(1, 59) = 7.413; *p* = 0.008; *ƞ*_*p*_^2^ = 0.11; observed power = 0.76) suggesting that low and high performers significantly differed with respect to VSCL scores regardless of Stimulation. More interestingly, a significant Stimulation*Performance interaction was found (*F*(4, 59) = 3.102; *p* = 0.022; *ƞ*_*p*_^2^ = 0.17; observed power = 0.78). Post-hoc analysis revealed that the P3 – 3 mA offline AtDCS group’s reduction in VSCL scores with respect to the Sham group was significant only for low performers (low performers: *p* = 0.006; high performers: *p* = 0.166; Fig. 5A). The P3 – 1.5 mA offline AtDCS group’s reduction in VSCL scores did not reach significance neither for the low nor for the high performers group (low performers: *p* = 0.136; high performers: *p* = 0.247) though this result could be due to a reduced statistical power consequent to the halving of the sample as low performers broadly revealed a greater interference with respect to high performers (Fig. 5B).

^(CF)^ A 5 × 13 mixed design ANCOVA with the between factor Stimulation (5 levels: P3 - 3 mA offline AtDCS, P3 - 3 mA online AtDCS, P3 - 1.5 mA offline AtDCS, P3 - 1.5 mA online AtDCS and Sham), the within factor Blocks (13 levels) and General Task Performance (i.e., mean accuracy scores on *new* trials) as covariate factor, was performed to evaluate the influence of different AtDCS protocols on VSCL. Mean hit trials for each stimulation group were: 0.84 (P3 - 3 mA offline AtDCS), 0.85 (P3 - 3 mA online AtDCS), 0.81 (P3 - 1.5 mA offline AtDCS), 0.83 (P3 - 1.5 mA online AtDCS) and 0.85 (Sham). The analysis revealed a significant main effect of Stimulation (*F*(4, 63) = 2.542; *p* = 0.048; *ƞ*_*p*_^2^ = 0.14; observed power = 0.69). Compared to the Sham group, a significant reduction of VSCL scores for the P3 – 3 mA offline AtDCS group (P3 – 3 mA offline AtDCS: 56.77 ms; Sham: 91.84 ms; *p* = 0.008) and a trend in the direction of a significant reduction for the P3 – 1.5 mA offline AtDCS group (P3 – 1.5 mA offline AtDCS: 68.92 ms; *p* = 0.074) was revealed by the post-hoc analysis (Figs. 3A and 4A). The covariate factor was also significant (*F*(1, 63) = 9.073; *p* = 0.004; *ƞ*_*p*_^2^ = 0.13; observed power = 0.84) suggesting, once again, an important influence of general task performance on VSCL scores. No other main effect or interactions were significant to the statistical analysis (all *ps* > 0.232).

**Table 1:** Reaction time (RT) for new or old arrays in low and high performers subjects separated for the eight stimulation protocols. VSCL = visuo-spatial contextual learning score.

	RT new	RT old	VSCL	low performers			median	high performers		
				RT new	RT old	VSCL		RT new	RT old	VSCL
P3 - 3 mA offline AtDCS	918.6	861.8	***56.8***	948.8	923.2	25.6	83%	892.6	809.2	83.5
P3 - 3 mA online AtDCS	926.0	848.6	***77.4***	936.8	852.4	84.5	82%	915.2	844.8	70.4
P3 - 1.5 mA offline AtDCS	952.4	883.5	***68.9***	960.8	910.5	50.4	77%	944.0	856.6	87.5
P3 - 1.5 mA online AtDCS	942.3	855.9	***86.3***	982.7	892.8	89.9	81%	901.9	819.1	82.8
P3 – Sham	919.7	827.8	***91.8***	942.9	866.7	76.2	81%	896.5	789.0	107.4
P4 - 3 mA offline AtDCS	935.9	878.7	***57.2***	968.0	925.2	42.9	77%	903.9	832.3	71.6
P3 - 3 mA offline CtDCS	915.0	830.2	***84.8***	941.3	857.2	84.1	80%	888.6	803.2	85.4
P3 - 3 mA online CtDCS	945.0	874.3	***70.7***	967.6	893.8	73.8	76%	922.3	854.7	67.6

**Figure 5:**
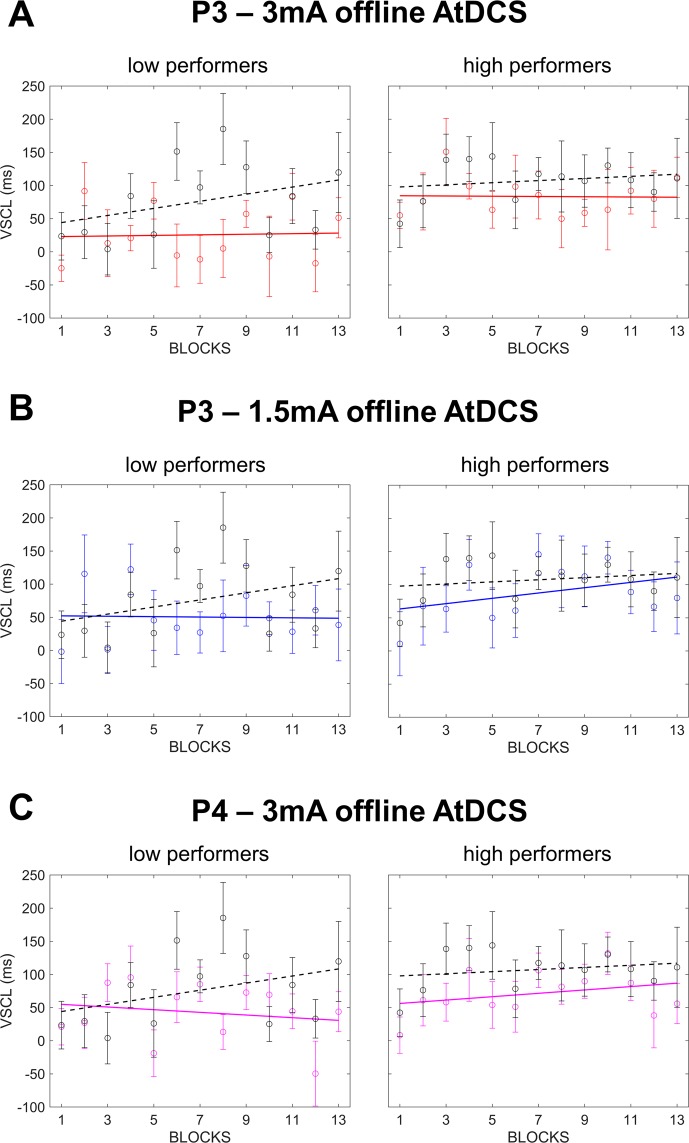
Linear fits of VSCL scores (i.e., RTs on *new* trials minus RTs on *old* trials) across blocks from three tDCS protocols of interest, i.e., P3 – 3 mA AtDCS (**A**), P3 – 1.5 mA AtDCS (**B**) and P4 – 3 mA AtDCS (**C**). Results are divided between low and high performers based on group accuracy scores’ median values on *new* trials (see Table 1 for details).

### Experiment 2 - right-PPC offline AtDCS

^(CF)^A 2 × 13 mixed design ANCOVA with the between factor Stimulation (2 levels: P4 - 3 mA offline AtDCS and Sham), the within factor Blocks (13 levels) and General Task Performance as covariate factor, was performed to evaluate the contribution of right-PPC in the reduction of VSCL scores reported in Experiment 1. The analysis compared data obtained from a stimulation protocol in which the target electrode was placed over P4 with data obtained during Sham stimulation administered in Experiment 1. Mean hit trials were: 0.81 (P4 - 3 mA offline AtDCS) and 0.81 (Sham). Results revealed a main effect of Stimulation (*F*(1, 25) = 5.604; *p* = 0.026; *ƞ*_*p*_^2^ = 0.18; observed power = 0.62). A significant reduction in VSCL scores was evident on P4 - 3 mA offline stimulation protocol (57.22 ms; *p* = 0.021) with respect to the Sham protocol (Sham: 91.84; Figs. 3B and 4B). The covariate factor was not significant (*F*(1, 25) = 1.907; *p* = 0.179; *ƞ*_*p*_^2^ = 0.07) and no other main effects or interactions were significant to the statistical analysis (all *ps* > 0.249).

^(EX)^ Also in this case, two 2 × 13 mixed ANOVAs were performed on accuracy scores and RTs to *new* trials with the between factor Stimulation (3 levels) and the within factor Blocks (13 levels) to control whether differences in VSCL scores could be attributable to between groups differences in general task performance. Both analyses revealed a main effect of Blocks (accuracy: *F*(12, 312) = 2.371; *p* = 0.006; *ƞ*_*p*_^2^ = 0.08; observed power = 0.96; RTs: *F*(12, 312) = 1.963; *p* = 0.027; *ƞ*_*p*_^2^ = 0.07; observed power = 0.91) explained by a general increase in accuracy and reduced RTs as the task progressed. Both the main effect of Stimulation (accuracy: *F*(1, 26) = 0.087; *p* = 0.771; *ƞ*_*p*_^2^ = 0.003; RTs: *F*(1, 26) = 0.429; *p* = 0.518; *ƞ*_*p*_^2^ = 0.01) and the Stimulation*Block interaction (accuracy: *F*(12, 312) = 0.770; *p* = 0.681; *ƞ*_*p*_^2^ = 0.03; RTs: *F*(12, 312) = 0.816; *p* = 0.633; *ƞ*_*p*_^2^ = 0.03) were not significant, revealing that the two stimulation groups were correctly equated with respect to general task performance (see also Appendix A in the Supplementary Information for further control analyses on this aspect).

^(EX)^Again, for explorative purposes, an additional analysis was conducted after dividing participants into “low performers” and “high performers” based on group accuracy scores’ median values on *new* trials (Table 1). A 2 × 2 × 13 mixed ANOVA with the between factors Stimulation (2 levels) and Performance (2 levels: low performers and high performers) and the within factor Blocks (13 levels) was performed on VSCL scores. Results revealed a main effect of Stimulation (*F*(1, 24) = 6.437; *p* = 0.018; *ƞ*_*p*_^2^ = 0.21; observed power = 0.68) that was explained by a significant reduction of VSCL scores on P4 - 3 mA offline stimulation protocol (*p* = 0.018) with respect to the Sham protocol. As expected, a main effect of Performance was evident (*F*(1, 24) = 4.818; *p* = 0.038; *ƞ*_*p*_^2^ = 0.17; observed power = 0.58). However, the interaction Stimulation*Performance was not significant (*F*(1, 24) = 0.008; *p* = 0.928; *ƞ*_*p*_^2^ = 0.0003) suggesting that stimulation affected VSCL on both low and high performers (Fig. 5C).

### Experiment 3 - left-PPC 3 mA CtDCS varying timings

^(CF)^ A 3 × 13 mixed design ANCOVA with the between factor Stimulation (3 levels: P3 – 3 mA offline CtDCS, P3 – 3 mA online CtDCS and Sham), the within factor Blocks (13 levels) and General Task Performance (i.e., mean accuracy scores on “new trials”) as covariate factor was performed to evaluate the effect of offline and online CtDCS on VSCL scores. Again, data from the Sham protocol were taken from Experiment 1. Mean hit trials were: 0.83 (P3 – 3 mA offline CtDCS), 0.81 (P3 – 3 mA online CtDCS) and 0.85 (Sham). The analysis revealed no significant main effects (all *ps* > 0.490) or interactions (all *ps* > 0.342) suggesting that the three stimulation protocols had similar effects on VSCL (Figs. 3C and 4C).

^(EX)^ Two 3 × 13 ANOVAs on accuracy scores and RTs on *new* trials were performed to ensure comparable performances between groups on the task. A main effect of Blocks was evident both for accuracy scores and RTs (accuracy: *F*(12, 468) = 2.024; *p* = 0.021; *ƞ*_*p*_^2^ = 0.05; observed power = 0.92; RTs: *F*(12, 468) = 1.98; *p* = 0.024; *ƞ*_*p*_^2^ = 0.05; observed power = 0.92) explained by increased accuracy and decreased RTs as the task progressed. Neither the main effect of Stimulation (accuracy: *F*(1, 39) = 0.743; *p* = 0.482; *ƞ*_*p*_^2^ = 0.04; RTs: *F*(1, 39) = 1.17; *p* = 0.321; *ƞ*_*p*_^2^ = 0.06), nor the Stimulation*Blocks interaction (accuracy: *F*(24, 468) = 0.606; *p* = 0.930; *ƞ*_*p*_^2^ = 0.03; RTs: *F*(24, 468) = 1.10; *p* = 0.339; *ƞ*_*p*_^2^ = 0.05) were significant to the statistical analysis, confirming that the three stimulation groups were correctly equated with respect to general task performance (see also Appendix A in the Supplementary Information for further control analyses on this aspect).

### Sensations questionnaire

^(EX)^ Wilcoxon Signed-Ranks Test was used to first evaluate whether sensations induced by online and offline Sham stimulations were statistically comparable^26,29^. Results revealed no statistical differences between online Sham ranks and offline Sham ranks for all the examined sensations (all *ps* > 0.422). Data from the offline and online questionnaires were then collapsed into a single Sham condition. For the other stimulation protocols, only sensations referring to the real (offline or online) stimulation were examined.

^(CF)^ The Kruskal-Wallis test was used to compare the different stimulations for each sensation. The analysis revealed no significant difference between the stimulation protocols for any of the examined sensation (all *ps* > 0.131) suggesting that sensations experienced by different stimulation groups were comparable (see Table 2 for a more detailed description of sensations induced by each stimulation protocol).

**Table 2:** Sensations values.

	Irritation	Pain	Burning	Heat	Itch	Iron Taste	Fatigue
P3 - 3 mA offline AtDCS	0.54	0.15	0.61	0.46	1.61	0.46	0.38
P3 - 3 mA online AtDCS	0.86	0.36	1.07	0.79	1.50	0.14	0.43
P3 - 1.5 mA offline AtDCS	0.71	0.36	0.64	0.93	1.21	0.00	0.21
P3 - 1.5 mA online AtDCS	0.57	0.50	0.50	0.64	0.86	0.14	0.57
P3 – Sham	0.64	0.36	0.75	0.32	1.00	0.39	0.54
P4 - 3 mA offline AtDCS	0.07	0.43	1.21	0.57	1.07	0.71	0.43
P3 - 3 mA offline CtDCS	0.64	0.21	0.71	0.71	1.50	0.43	0.79
P3 - 3 mA online CtDCS	0.36	0.29	0.29	0.14	1.07	0.14	0.50

Mean value of each reported sensation separated for the eight stimulation protocols.

### General remarks on the 3 mA stimulation protocols

We would like to point out that some of the tDCS protocols administered in the present study included the use of current intensities higher than those generally administered in previous works (usually not exceeding 1-2 mA). The reason we decided to use such intensities is twofold. On the one hand, we aimed at testing the possibility to induce more robust behavioural outcomes as a consequence of stronger electric fields targeting the brain^35,36^. On the other hand, we aimed at examining the effective tolerability of such intensities in a large group of healthy participants^26,29^. The robustness of the produced behavioural outcome could be partly assumed by the internal replication of our main finding (i.e., strong VSCL reduction after 3 mA offline AtDCS in both Experiment 1 and 2) though we cannot exclude replication also with lower intensities. Regarding tolerability aspects, we found that even higher stimulation intensities (3 mA) were well tolerated by participants who reported sensations similar to those induced by lower intensities (1.5 mA) and that were also mostly indistinguishable from those evoked by the control Sham protocol. From the numerical point of view, the augmentation from 1.5 to 3 mA led to an increase of the means of the aggregate sensations from 0.58 to 0.62 (P3 - and P4 – 3 mA offline AtDCS conditions collapsed) for the offline AtDCS conditions (0.71 for the CtDCS) and from 0.53 to 0.73 for the online AtDCS conditions (0.39 for the CtDCS). The aggregate sensations from the Sham condition was 0.57. Even when considering the most reported sensations (Burning and Itch; see Table 2), the increase in stimulation intensity did not produce substantial changes and scores remained into a tolerability range. For Burning, the enhancement from 1.5 to 3 mA led to an increase from 0.64 to 0.91 (P3 - and P4 – 3 mA offline AtDCS conditions collapsed) for the offline AtDCS conditions (0.71 for the CtDCS) and from 0.50 to 1.07 for the online AtDCS conditions (0.29 for the CtDCS) with Sham at 0.75. For Itch, the enhancement from 1.5 to 3 mA led to an increase from 1.21 to 1.34 (P3 - and P4 – 3 mA offline AtDCS conditions collapsed) for the offline AtDCS conditions (1.50 for the CtDCS) and from 0.86 to 1.50 for the online AtDCS conditions (1.07 for the CtDCS) with Sham at 1.00.

In general, only few subjects reported scores equal or higher than 3 (3 = “I felt the described sensation to a considerable degree”; 4 = “I strongly felt the described sensation”). Participants who reported scores equal or higher than 3 for Burning were 2 out of 27 in the 3 mA offline AtDCS (P3 - and P4 – 3 mA offline AtDCS conditions collapsed), 2 out of 14 in the 3 mA online AtDCS and 0 for all the other stimulation protocols. Similarly, participants reporting scores equal or higher than 3 for Itch were 2 out of 27 in the 3 mA offline AtDCS (P3 - and P4 – 3 mA offline AtDCS conditions collapsed), 5 out of 14 in the 3 mA online AtDCS, 1 out of 14 in the 3 mA offline CtDCS, 1 out of 14 for the 3 mA online CtDCS, 0 out of 14 in the 1.5 mA offline AtDCS and 1 out of 14 in the 1.5 mA online AtDCS. Crucially, no participant reported relevant cutaneous or other adverse effect.

## Discussion

In the present study, we used a series of tDCS protocols to test the effects of various parameters of application in shaping VSCL. We provided evidence of relevant tDCS-brain interactions in both left and right PPC possibly related to homeostatic regulatory mechanisms similar to those originally observed in the motor cortex^14,20,37–39^.

In Experiment 1, we showed that AtDCS applied on left-PPC before the execution of the task strongly reduced VSCL with respect to the control Sham protocol. This was evident both for the 3 mA and for the 1.5 mA AtDCS, though a more robust reduction was produced by the 3 mA protocol. This result was not due to the influence of tDCS on general task performance or response speed, as no differences between stimulation protocols were made evident when the sole performances on *new* trials were considered^40^. Furthermore, it is unlikely that this result could be attributed to differences in the sensations induced by the stimulation protocols as the analysis of the sensations questionnaires revealed statistically comparable results between all the stimulation protocols administered. Finally, this result unlikely arose from a reduced activation of the right PPC consequent to a tDCS-induced alteration of the interhemispheric rivalry between left and right PPC^41^. Given the prominent role of the right-PPC in visuo-spatial processing^42,43^, an alternative interpretation could consider a tDCS-induced hyperactivation of the left PPC and a consequent hypoactivation of the right PPC producing the VSCL reduction here reported. However, if it was the case, we would expect that applying the same stimulation protocol over the right-PPC would lead to an increase of VSCL. We tested this hypothesis in Experiment 2, where the stimulation protocol that produced the more robust interference in Experiment 1 (i.e., P3 – 3 mA offline AtDCS) was used with a reversed electrodes montage (i.e., target electrode on P4 and return electrode on the left arm) and results revealed overlapping effects with those obtained after left PPC intervention, suggesting an equal contribution of left and right PPC in VSCL.

We concluded that PPC offline AtDCS selectively influenced the ability of participants to store new visuo-spatial contextual information. A plausible explanation is that the interaction of two excitability-increasing events (i.e., AtDCS and VSCL) hindered the normal task-related neuroplastic phenomena. As conceptualized by an influential model of synaptic plasticity elaborated by Bienenstock, Cooper and Munro model (BCM model), the stabilization of neuronal activity from an excessive increase or decrease in neuronal firing rate would be ensured by the dynamic adaptation of the modification threshold (*θ*_*m*_), that is the level of post-synaptic activity above or below which LTP or LTD are respectively induced^44^. In this view, the prolonged excitatory input produced by AtDCS could have led to an increase in the modification threshold producing a consequent decrease in the probability of subsequent excitatory events (i.e., VSCL) to induce LTP.

Contrary, when AtDCS was applied during the execution of the task no significant changes in VSCL were produced as compared to the Sham group. This result is in line with a recent study reporting no behavioural changes induced by AtDCS applied during the execution of the same task^24^. However, given the plausible strong PPC activation resulting from the two concurrent excitability-increasing events (i.e., AtDCS and VSCL), one might wonder why no homeostatic mechanisms had been observed in this case. One possibility is that AtDCS produced an increase of the modification threshold only when VSCL had already occurred and thus did not influence its occurrence. Indeed, homeostatic mechanisms are not instantaneous and are known to develop after few or tens of minutes of prolonged plasticity-inducing protocols^45^. Furthermore, the length of breaks intervening between the two events (i.e., AtDCS and VSCL) could have played a relevant role. In this regard, Fricke and colleagues reported that homeostatic plasticity in the motor cortex occurred only when 3 or 10 minutes breaks intervened between the two activity enhancing protocols, while no homeostatic mechanisms were observed for both longer and shorter breaks^46^. In the offline conditions, the time lapse between AtDCS and VSCL ideally matched with those reported by Fricke and colleagues whereas the same does not hold true for the online conditions. Another interesting point to be examined concerns the lack of a behavioural facilitation expected to occur in the online AtDCS conditions^14,20^. One possibility is that some sort of ceiling/floor effect intervened in our case, as VSCL in the control Sham protocol was overall high (mean: 91.8 ms; Table 1) with respect to VSCL reported in previous works (e.g., Chun and colleagues ~58 ms^25^, Nydam and colleagues ~50 ms^24^) and possibly no intervention could have been able to further enhance it. At the same time, we cannot a priori assume a sharp direct relationship between online PPC AtDCS application and VSCL increase given that a distributed network of cortical activity is involved in this type of learning.

**Figure 6:**
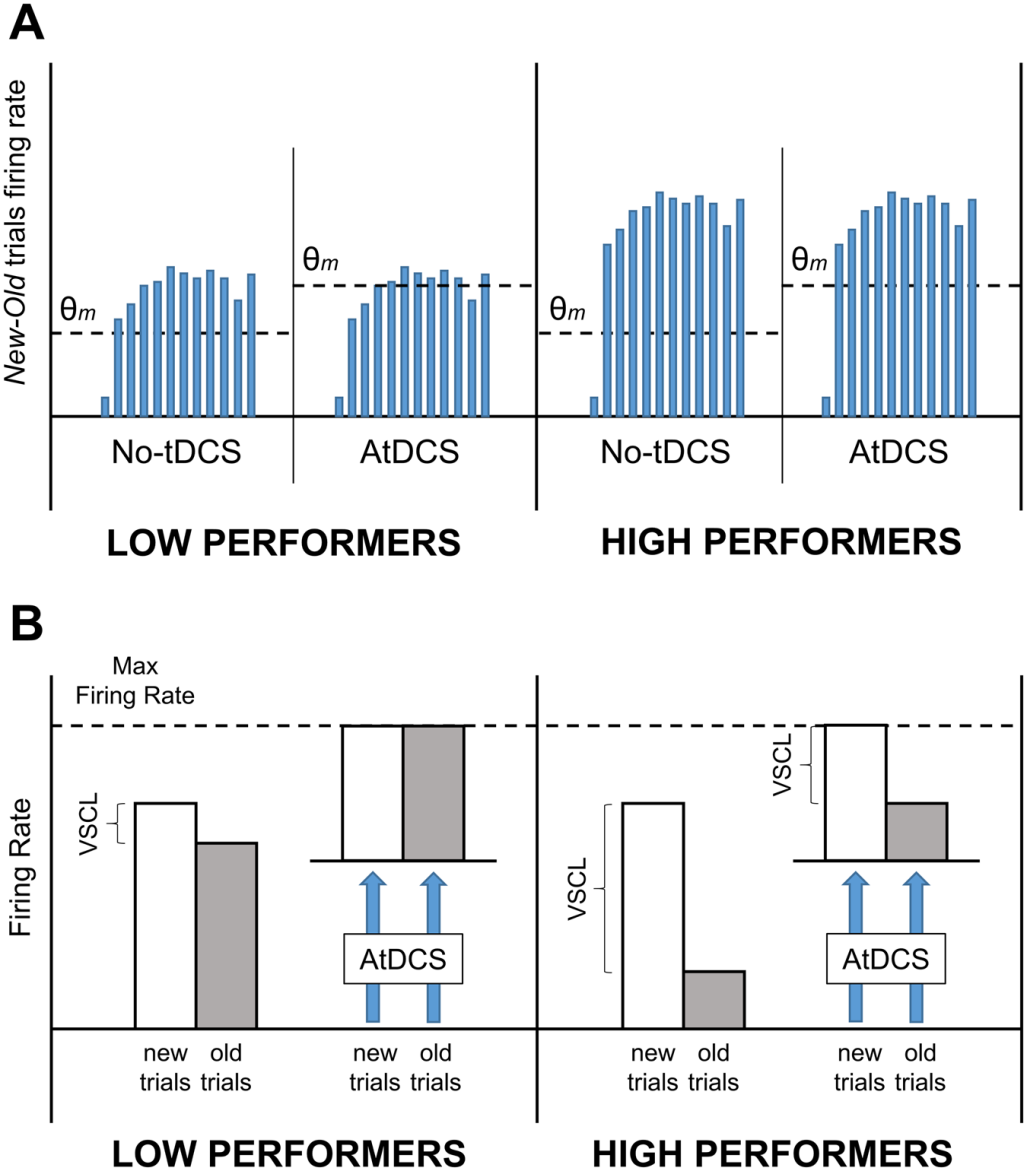
Graphical representation of the two interpretative hypotheses of the results. **A**. A prolonged exposure to AtDCS would have raised the modification threshold (*θ*_*m*_) reducing the probability of learning of the repeated visuo-spatial contexts. Bars represent the hypothetical difference between neuronal firing rate produced by *new* trials presentation and *old* trials presentation across the 13 blocks (each bar a block). Low performers would be more affected because of a general reduced difference in neuronal firing rate responses to *new* and *old* trials. **B**. Low performers would generally show a reduced difference between neuronal firing rate produced by *new* and *old* trials presentation with respect to high performers. This difference would be cancelled in low performers while still preserved in high performers after the indiscriminate increase in neuronal firing rate produced by AtDCS.

At the neural level, the spatial contextual cueing paradigm has been associated with a pattern of multifaceted cortical responses including primary visual areas, parietal and frontal cortices as well as various medial temporal lobe structures^22–24,31–33,47^. Some studies reported that *old* trials presentation was associated with a reduced activation of these areas likely reflecting a more fluid processing of the stored visual scenes^22,23,32,33,47^. Conversely, other studies showed an enhanced activation interpreted as a memory-mediated attentional capture of repeated spatial contexts^31–33^. It is possible that both mechanisms are at play. In this regard, Manginelli and colleagues showed that different temporo-parietal areas were associated with opposite activations during *old* trials presentation^33^. However, regardless of the exact neural mechanisms at play, it seems clear that distinct neuronal activity levels in response to *new* and *old* trials are an important correlate of VSCL. Given the direct relationship found between the ability to perform the task (as assessed by general task performance measure; i.e., mean accuracy scores on not repeated trials) and VSCL scores, we could predict high performers to show a greater difference in the neural activation produced by *new* and *old* trial with respect to low performers. If it was the case, the different pattern of results obtained by low and high performers reported in Experiment 1 could be explained in the light of two possible interpretations. The first interpretation would consider the raising up of the modification threshold as the main mechanism at play. In this view, those subjects showing higher differences in neural activation produced by *new* and *old* trials presentation (i.e., high performers) would still be able to exceed modification threshold and achieve VSCL. This would not happen for low performers whose VSCL would be mostly cancelled after the raising up of the modification threshold (Fig. 6A). The second interpretation would mainly consider the interaction between task-induced and tDCS-induced neural noise^48^. In this view, AtDCS could have produced a boosted neuronal activity leading PPC neurons close to their saturation level and consequently reducing (or even cancelling) the difference between *new* and *old* trials-induced activation in low performers. The same mechanism could have only marginally affected high performers for whom the difference between *new* and *old* trials-induced activation would have been mostly preserved (Fig. 6B). However, though potentially valid, the latter interpretation would not fully explain why similar mechanisms were not retrievable in the online conditions. We then consider the former interpretation as the more plausible of the two.

Despite this interpretation, it remains to be clarified why in Experiment 2 the difference between low and high performers was not confirmed and only a main effect of Stimulation was evidenced by the statistical analysis. It is possible that this was due to differences in how participants were assigned to low and high performers groups as the median accuracy values of general task performance were slightly different between the two stimulation groups (P3 - 3 mA AtDCS: 83.3%; P4 - 3 mA AtDCS: 76.9%; Table 1). Anyway, the present experiment was not developed to fully investigate the interactions between tDCS and general task performance, thus we acknowledge interpretations of this aspect are somehow limited with the present data.

Experiment 3 was designed to take a further step in the direction of disclosing homeostatic principles in the PPC. We probed for facilitatory behavioural effects after offline and online application of 3 mA CtDCS to the left PPC. Results revealed that CtDCS produced no significant changes in VSCL with respect to the control Sham protocol neither when applied before nor when applied during task execution. Though to some extent inconsistent with the BCM model, the lack of a significant effect of the offline CtDCS protocol on VSCL could be explained in several ways. On the one hand, we could again hypothesize that a ceiling/floor effect intervened or that CtDCS induced small changes that were not retrievable at the behavioural level. On the other hand, a sharp inverse relationship between AtDCS and CtDCS-induced behavioural changes cannot be a priori assumed given the non-linearity effects of tDCS^12^. It could be argued that higher or lower CtDCS stimulation intensities are potentially necessary to reverse the behavioural effect produced by AtDCS. What is maybe more surprising is the null effect of the online CtDCS. We expected this protocol to produce a significant reduction of VSCL like the one observed after offline AtDCS. Nevertheless, the lack of a symmetrical relationship between the effects produced by AtDCS and CtDCS have been reported in various studies. For example, in a meta-analytical review Jacobson and collaborators^2^ reported that the relationship anodal-excitatory and cathodal-inhibitory have been mainly shown within motor and sensory domains. Conversely, when higher cognitive functions are involved, findings are much more controversial and a lack of significant behavioural modifications after CtDCS are frequently evidenced. Though it is still debated what is the reason for this asymmetry within higher cognitive domains, various explanations have been proposed. These mainly pertain the recruitment of larger cortical networks, mechanisms of contralateral compensation and the initial activation state of the brain^2^. In his view, the not-significant change produced by CtDCS intervention in our study could be attributed to one of the abovementioned mechanisms. Furthermore, given that online effects of tDCS are thought to primarily affect resting membrane potential while offline effects to be dependent on synaptic plasticity^1^, we can hypothesize that plasticity-mediated interference has a much stronger impact on behaviours with respect to changes in resting state potentials.

## Conclusions

In summary, the present study revealed that the exposure to a prolonged excitability increasing stimulation of the PPC reduces VSCL when applied before task execution but left VSCL unaffected when applied during task execution. We consider the present results relevant in the context of a deeper understanding of the effects produced by tDCS in brain regions outside the motor cortex together with the potential interactions of these effects with other plasticity inducing events.

## Supplementary information

Supplementary information

## Data Availability

The data sets generated during the current study are available from the corresponding authors on rational request.
