# Supplementary material for: Effects of different transcranial direct current stimulation protocols on visuo-spatial contextual learning formation: evidence of homeostatic regulatory mechanisms
Source: Sci Rep. 2020 Mar 12;10:4622. doi: 10.1038/s41598-020-61626-7 (PMC7067887; doi:10.1038/s41598-020-61626-7)
Supplement: Supplementary file 1 — Supplementary information [file 41598_2020_61626_MOESM1_ESM.docx]

**Effects of different transcranial direct current stimulation protocols on visuo-spatial contextual learning formation: evidence of homeostatic regulatory mechanisms**

Paolo A. Grasso^1^*, Elena Tonolli^1^, Carlo Miniussi^1^*

^1^Centre for Mind/Brain Sciences - CIMeC, University of Trento, Rovereto (TN), Italy

**Appendix A. Analyses on Training Blocks**

*Analysis*

To further control for comparable performances across groups on general task performance, different ANOVAs with the between factor Stimulation (5 levels in Experiment 1, 2 levels in Experiment 2 and 3 levels in Experiment 3) and the within factor Blocks (3 levels) were conducted on performances (i.e., RTs and Accuracy) obtained on Training Blocks. A p-value < 0.05 was considered significant for all statistical analyses.

*Results*

Experiment 1: ^(CF)^ The main effect of Stimulation and the interaction Stimulation*Blocks were far from significance for both RTs (Stimulation: *F*(4, 64) = 0.57; *p =* 0.688; *ƞ_p_^2^* = 0.03; Stimulation*Blocks: *F*(8,128) = 1.00; *p =* 0.442; *ƞ_p_^2^* = 0.06) and Accuracy scores (Stimulation: *F*(4, 64) = 0.376; *p =* 0.825; *ƞ_p_^2^* = 0.02; Stimulation*Blocks: *F*(8,128) = 1.208; *p =* 0.299; *ƞ_p_^2^* = 0.07) confirming equated performances across groups.

Experiment 2: ^(CF)^ Again, neither the main effect of Stimulation nor the interaction Stimulation*Blocks were significant on both RTs (Stimulation: *F*(1, 26) = 0.526; *p =* 0.475; *ƞ_p_^2^* = 0.02; Stimulation*Blocks: *F*(2,52) = 0.525; *p =* 0.595; *ƞ_p_^2^* = 0.02) and Accuracy scores (Stimulation: *F*(1, 26) = 0.110; *p =* 0.742; *ƞ_p_^2^* = 0.004; Stimulation*Blocks: *F*(2,52) = 1.468; *p =* 0.240; *ƞ_p_^2^* = 0.05).

Experiment 3: ^(CF)^ The main effect of Stimulation and the interaction Stimulation*Blocks were not significant for RTs (Stimulation: *F*(2,39) = 1.48; *p =* 0.241; *ƞ_p_^2^* = 0.07; Stimulation*Blocks: *F*(4,78) = 0.51; *p =* 0.730; *ƞ_p_^2^* = 0.02) while a main effect of Stimulation was evident on Accuracy scores (*F*(2,39) = 3.712; *p =* 0.033; *ƞ_p_^2^* = 0.16) mainly explained by reduced accuracy for the P3 – 3 mA online CtDCS group (mean: 0.65) with respect to both the P3 – 3 mA offline CtDCS (mean: 0.76) and the Sham (0.71) groups. The interaction Stimulation*Blocks was instead not significant (*F*(4,78) = 0.329; *p =* 0.857; *ƞ_p_^2^* = 0.02).

*Conclusions*

These analyses confirmed once again that the different groups had statistically comparable performances on visual search ruling out the possibility that results from Experiment 1 and 2 could be explained by initial between groups’ differences in general task performance. Nevertheless, in order to fully control for this aspect, we consider more appropriate the analysis employed on the sole *new* trials reported in the Results section of the manuscript. Indeed, on the one hand training blocks were mainly meant to make participants familiarize with the task and a greater performance variability is somehow expected with respect to performances obtained during the experimental session. On the other hand, training blocks were always performed ~18 minutes before experimental session, a period during which learning processes could have occurred.

**Appendix B. Analyses on Transcranial Random Noise Stimulation (tRNS) protocols**

The full study also comprised four additional tRNS protocols administered in a different group of participants. We here report the main results alongside with methodological information about the stimulation protocols and the procedure used.

*Participants*

The procedure for the selection of participants was exactly the same employed for the tDCS protocols (see *Participants* section in the manuscript for further details) which led to the selection of fifty-six healthy participants (mean age: 22.6 years, sd: 3.02 years, 28 males) naïve to the purpose of the study, right handed and with normal or corrected to normal visual acuity. Participants were pseudo-randomly assigned to one of the four electrical stimulation protocols (see *Transcranial Random Noise Stimulation* section below) which resulted in the selection of 14 participants (7 males) per group. Before taking part to the experiment, all participants were informed about the procedures of the study and provided written informed consent.

*Transcranial Random Noise Stimulation*

The equipment for tRNS (i.e. stimulator, electrodes, sponges and electro-conductive gel) was the same used for tDCS (see *Transcranial Direct Current Stimulation* section in the manuscript for further details). The smaller electrode (i.e. 5 x 5 cm) was always placed on the left-PPC (i.e. P3 of the 10/20 EEG system) while the other electrode (i.e. 6 x 7 cm) was positioned on the right arm. The current was a high frequency (101-640 Hz) random noise pattern delivered at intensities of either 3 or 1.5 mA (peak to peak) and applied for 15 minutes. Four different stimulation protocols were administered (i.e. 3 mA offline tRNS, 3 mA online tRNS, 1.5 mA offline tRNS and 1.5 online tRNS). In the offline protocols real stimulation was applied before the execution of the task while a sham stimulation was delivered during the task. Conversely, in the online protocols real stimulation was applied during the execution of the task while a sham stimulation was delivered before the task. Sham stimulation consisted of a direct current stimulation applied in the first and last 20s (i.e., 10s ramped up and 10s ramped down).

*Analysis*

VSCL scores (i.e., RTs on *new* trials minus RTs on *old* trials) were used as the main outcome measure. Only hit trials and trials with VSCL scores within 3sd from the individual mean were considered in the analysis. Data were analyzed using a mixed design analysis of covariance (ANCOVA) comparing the effects of different stimulation protocols on VSCL scores after controlling for general task performance (covariate factor; i.e., mean accuracy scores on *new* trials). VSCL from the Sham group collected in Experiment 1 was also included in the analysis. A p-value < 0.05 was considered significant for all statistical analyses.

*Results*

^(CF)^ A 5 x 13 mixed design ANCOVA with the between factor Stimulation (5 levels: 3 mA offline tRNS, 3 mA online tRNS, 1.5 mA offline tRNS, 1.5 online tRNS and Sham), the within factor Blocks (13 levels) and General Task Performance (i.e., mean accuracy scores on *new* trials) as covariate factor, was performed to evaluate the influence of different tRNS protocols on VSCL. The analysis revealed that both the main effect of Stimulation (*F*(4, 64) = 1.733; *p =* 0.153; *ƞ_p_^2^* = 0.10) and the interaction Stimulation*Blocks (*F*(48, 768) = 1.197; *p =* 0.173; *ƞ_p_^2^* = 0.07) were not significant suggesting no relevant influence of tRNS on VSCL. Also, the main effect of Blocks was not significant (*F*(12,768) = 1.407; *p =* 0.157; *ƞ_p_^2^* = 0.02) while the covariate factor showed a trend toward significance (*F*(1, 64) = 3.411; *p* = 0.069; *ƞ_p_^2^* = 0.05).

*Conclusion*

Our results showed that tRNS was not capable to significantly modulate VSCL. Both the intensity (3 mA and 1.5 mA) and the timing of application (offline and online) did not produce significant changes in VSCL with respect to the condition of Sham stimulation.

**Appendix C. Analyses at the 1-week Follow-up session**

The study also aimed at investigating the presence of long-lasting effects of tES on VSCL. For this reason, participants were retested at a 1-week Follow-up session with the same experimental task but without the use of electrical stimulation. Results are reported below separately for each experiment.

*Participants*

Analysis at the 1-week Follow-up session comprised one hundred and sixty-four participants out of a total of one hundred and sixty-eight taking part to the first experimental session. Indeed, three participants withdrew after the first session (two assigned to the P4 – 3 mA offline AtDCS and one to the P3 – 3 mA online CtDCS) and one participant, assigned to the P3 – 3 mA offline AtDCS, was excluded because outlier in the first session (see *Multivariate Outliers’ Detection* section in the manuscript).

*Analysis*

VSCL scores (i.e., RTs on *new* trials minus RTs on *old* trials) were used as the main outcome measure. Only hit trials and trials with VSCL scores within 3sd from the individual mean were considered in the analysis. Data were analyzed using a mixed design analysis of covariance (ANCOVA) with the between factor Stimulation (5 levels in Experiment 1; 2 levels in Experiment 2; 3 levels in Experiment 3; 5 levels in tRNS Experiment), the within factor Blocks (13 levels) and General Task Performance (i.e., mean accuracy scores on *new* trials) as covariate factor. Fisher’s Least Significant Difference (LSD) method was used to test the limited number of contrasts of interest (i.e., compare performances of groups receiving real stimulation with the performance of the group receiving sham stimulation). For all the other comparisons, Bonferroni correction was applied. A p-value < 0.05 was considered significant for all statistical analyses.

*Results*

Experiment 1: ^(CF)^ Results did not reveal neither a main effect of Stimulation (*F*(4, 63) = 1.184; *p =* 0.326; *ƞ_p_^2^* = 0.07) nor an interaction Stimulation*Blocks (*F*(48,756) = 0.797; *p =* 0.836; *ƞ_p_^2^* = 0.05) suggesting the lack of long-lasting effects of tDCS-induced modification of VSCL reported at the first session. The main effect of Blocks was not significant (*F*(12,756) = 1.365; *p =* 0.177; *ƞ_p_^2^* = 0.02) while the covariate factor was significant (*F*(1, 63) = 12.347; *p* < 0.001; *ƞ_p_^2^* = 0.16).

Experiment 2: ^(CF)^ Again, neither the main effect of Stimulation (*F*(1, 23) = 0.229; *p =* 0.636; *ƞ_p_^2^* = 0.01) nor the interaction Stimulation*Blocks (*F*(12,276) = 0.505; *p =* 0.911; *ƞ_p_^2^* = 0.02) were significant. The main effect of Blocks showed was not significant (*F*(12, 276) = 1.475; *p =* 0.133; *ƞ_p_^2^* = 0.06) while the covariate factor was significant (*F*(1, 23) = 12.347; *p* = 0.002; *ƞ_p_^2^* = 0.35).

Experiment 3: ^(CF)^ The main effect of Stimulation was not significant (*F*(2, 37) = 0.144; *p =* 0.867; *ƞ_p_^2^* = 0.007) while the interaction Stimulation*Blocks was significant (*F*(24,444) = 1.607; *p =* 0.035; *ƞ_p_^2^* = 0.08). The main effect of Blocks showed a trend towards significance (*F*(12,444) = 1.607; *p =* 0.086; *ƞ_p_^2^* = 0.04) and the covariate factor was significant (*F*(1, 37) = 4.526; *p* = 0.040; *ƞ_p_^2^* = 0.11).

tRNS Experiment: ^(CF)^ Neither the main effect of Stimulation (*F*(4,63) = 0.631; *p =* 0.642; *ƞ_p_^2^* = 0.04) nor the interaction Stimulation*Blocks (*F*(48,756) = 1.272; *p =* 0.106; *ƞ_p_^2^* = 0.07) were significant. Further the main effect of Blocks showed a trend towards significance (*F*(12,756) = 1.575; *p =* 0.094; *ƞ_p_^2^* = 0.02) while the covariate factor was not significant (*F*(1, 63) = 0.787; *p =* 0.378; *ƞ_p_^2^* = 0.01).

*Conclusions*

Our results revealed that the effects of stimulation evidenced at the first session were not retrievable at the 1-week Follow-up. One possibility is that tDCS-induced homeostatic mechanisms described in Experiment 1 and Experiment 2 did not produce a complete cancellation of VSCL (as also evident from our behavioral data) but rather led to a reduction of learning that did not abolish long-range consolidation mechanisms.
